# Antiviral Properties of the LSD1 Inhibitor SP-2509

**DOI:** 10.1128/JVI.00974-20

**Published:** 2020-09-15

**Authors:** Mitchell R. Harancher, Jessica E. Packard, Shane P. Cowan, Neal A. DeLuca, Jill A. Dembowski

**Affiliations:** a Department of Microbiology and Molecular Genetics, University of Pittsburgh School of Medicine, Pittsburgh, Pennsylvania, USA; b Department of Biological Sciences, Duquesne University, Pittsburgh, Pennsylvania, USA; University of Arizona

**Keywords:** DNA replication, HSV-1, herpes simplex virus, LSD1, SP-2509, antiviral

## Abstract

Lysine-specific demethylase 1 (LSD1) targets cellular proteins, including histone H3, p53, E2F, and Dnmt1, and is involved in the regulation of gene expression, DNA replication, the cell cycle, and the DNA damage response. LSD1 catalyzes demethylation of histone H3K9 associated with herpes simplex virus 1 (HSV-1) immediate early (IE) promoters and is necessary for IE gene expression, viral DNA replication, and reactivation from latency. We previously found that LSD1 associates with HSV-1 replication forks and replicating viral DNA, suggesting that it may play a direct role in viral replication or coupled processes. We investigated the effects of the LSD1 inhibitor SP-2509 on the HSV-1 life cycle. Unlike previously investigated LSD1 inhibitors tranylcypromine (TCP) and OG-L002, which covalently attach to the LSD1 cofactor flavin adenine dinucleotide (FAD) to inhibit demethylase activity, SP-2509 has previously been shown to inhibit LSD1 protein-protein interactions. We found that SP-2509 does not inhibit HSV-1 IE gene expression or transcription factor and RNA polymerase II (Pol II) association with viral DNA prior to the onset of replication. However, SP-2509 does inhibit viral DNA replication, late gene expression, and virus production. We used EdC labeling of nascent viral DNA to image aberrant viral replication compartments that form in the presence of SP-2509. Treatment resulted in the formation of small replication foci that colocalize with replication proteins but are defective for Pol II recruitment. Taken together, these data highlight a potential new role for LSD1 in the regulation of HSV-1 DNA replication and gene expression after the onset of DNA replication.

**IMPORTANCE** Treatment of HSV-1-infected cells with SP-2509 blocked viral DNA replication, gene expression after the onset of DNA replication, and virus production. These data support a potential new role for LSD1 in the regulation of viral DNA replication and successive steps in the virus life cycle, and further highlight the promising potential to utilize LSD1 inhibition as an antiviral approach.

## Abstract (precis)

Treatment of HSV-1-infected cells with SP-2509 blocked viral DNA replication, gene expression after the onset of DNA replication, and virus production. These data support a potential new role for LSD1 in the regulation of viral DNA replication and successive steps in the virus life cycle, and further highlight the promising potential to utilize LSD1 inhibition as an antiviral approach.

## INTRODUCTION

Herpes simplex virus 1 (HSV-1) is a prevalent alphaherpesvirus that infects the majority of the human population (1). Productive infection occurs in epithelial tissue and includes a series of events that occur on viral DNA, initiating immediately after entry into the nucleus. In the nucleus, viral genes are expressed through a temporal cascade of immediate early (IE) α, early (β), and late (γ) classes of genes (2, 3). Initial recruitment of transcription factors to IE genes is facilitated by the viral tegument protein VP16 (4). This mediates the expression of IE genes, which include infected cell polypeptides (ICPs) 0, 4, 22, 27, and 47. ICP4, the major viral transcription factor, binds to the viral DNA and recruits host transcription factors to promote the activation of early viral genes (5–7). These include genes that code for the viral DNA replication machinery. Viral genome replication, as well as ICP4 binding, mediates transcription of late viral genes. Late genes are classified as leaky late (γ1) or true late (γ2), depending on whether their expression is amplified or licensed by viral DNA replication, respectively. Furthermore, viral DNA replication is coupled to DNA recombination and repair (8). During DNA replication, intragenomic homologous recombination occurs between inverted repeats resulting in the formation of four genome isomers (9). Furthermore, intergenomic recombination occurs between coinfecting HSV-1 genomes (10–13), driving herpesvirus evolution (14). All of this is coordinated on the viral DNA in such a manner that initial infection through packaging of viral DNA into new capsids can occur in as little as 6 h (7).

HSV-1 also infects the innervating neurons of the peripheral nervous system, where it establishes a latent infection and viral gene expression is repressed (15). In the nuclei of neurons, viral DNA is packed into chromatin and there persists for the lifetime of the host. In response to stress to the host, HSV-1 periodically reactivates from latency to produce new virus particles. The mechanisms that mediate the removal of repressive chromatin from latent genomes are not completely understood, but it is clear that the state of the viral DNA is dynamic and can respond to cellular changes (16, 17).

On the contrary, HSV-1 genomes are largely nucleosome free after the onset of lytic gene expression (18–22) and all viral genes are equally accessible to micrococcal nuclease digestion regardless of their transcriptional state (23). Viral DNA is therefore accessible for increased interactions with DNA binding proteins relative to host chromatin (24). Therefore, although chromatin may affect the initial transcriptional competence of the HSV-1 genome, the temporal expression of viral genes is more likely regulated by transcription factor binding to the promoters of individual genes. For example, ICP4 preferentially binds to nucleosome-free viral DNA (25) and, through protein-protein interactions (5, 6), recruits cellular transcription factors (7, 25). ICP4 facilitates the activation of early genes and DNA replication is additionally required to license ICP4-mediated recruitment of transcription factors to late genes. How DNA replication is coupled to the activation of late viral gene expression is not completely understood.

Through proteomics of viral genome-associated proteins, we found that the cellular demethylase lysine-specific demethylase 1 (LSD1) associates with replicating viral DNA and viral replication forks (22, 26). LSD1 is a lysine specific demethylase that is well characterized for its role in the demethylation of H3K4 to repress cellular transcription (27). Another neuron-specific form of LSD1 demethylates histone H3K9 to promote transcription of target genes (28–30). LSD1 can therefore function as a repressor or activator of cellular transcription. LSD1 also demethylates nonhistone proteins, including p53, E2F, and Dnmt1 (31), and plays a role in replication fork pausing in fission yeast (32). LSD1 utilizes flavin adenine dinucleotide (FAD) as a cofactor for demethylation and therefore can be inhibited by the monoamine oxidase inhibitor (MAOI) tranylcypromine (TCP) and its derivative OG-L002, which covalently attach to and inactivate FAD. TCP and OG-L002 block viral reactivation from latency in animal models (33–35), consistent with an important role for LSD1 in productive infection. Furthermore, during lytic replication, LSD1 knockdown and inhibition by MAOIs cause increased levels of methylated histone H3K9 on the promoters of IE genes, resulting in a block in IE gene expression, DNA replication, and virus production in cell culture (33, 35). Therefore, LSD1 inhibition is an attractive target for HSV-1 antiviral therapy.

While investigating the effects of LSD1 inhibitors on HSV-1 infection, we recognized that inhibition by the small molecule SP-2509 does not block IE gene expression, early transcription factor association with viral genomes, early gene expression, or initiation of viral DNA replication. However, SP-2509 does block ongoing viral DNA replication, recruitment of RNA polymerase II (Pol II) to viral DNA after the onset of replication, and replication-dependent viral gene expression, resulting in strong inhibition of virus production. SP-2509 is a reversible LSD1 inhibitor that blocks LSD1 demethylase activity and interferes with LSD1 protein interactions (36, 37). Therefore, although both OG-L002 and SP-2509 inhibit HSV-1 infection, they may do so through different mechanisms. This study highlights a potential new role for LSD1 in the regulation of viral DNA replication and downstream steps in the HSV-1 life cycle and further highlights the promising potential to utilize LSD1 inhibition as an antiviral approach.

## RESULTS

### SP-2509 is a novel and potent inhibitor of HSV-1 infection.

**FIG 1:**
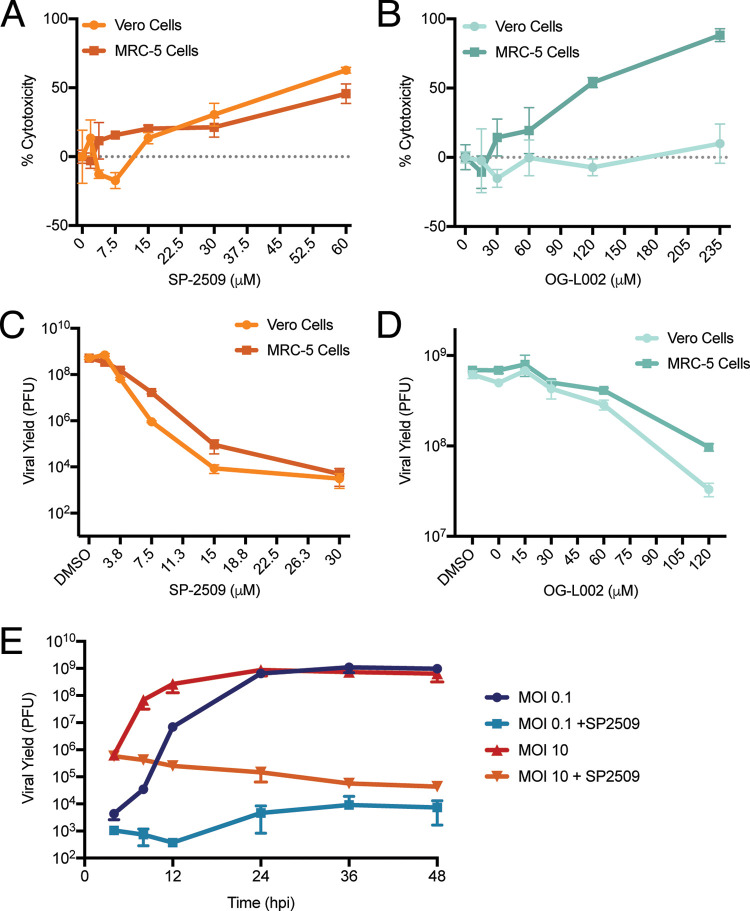
SP-2509 inhibits viral infection. (A and B) MTT cell proliferation assays (Abcam) were carried out to measure the cytotoxic effects of SP-2509 (A) and OG-L002 (B) on MRC-5 and Vero cells. Cells were incubated in the presence of indicated concentrations of inhibitor for 24 h before conducting the assay according to the manufacturer’s protocol. Data represent the mean of biological triplicate experiments with standard deviation. (C and D) Effects of SP-2509 (C) and OG-L002 (D) on HSV-1 viral yield were determined by infecting 1 × 10^6^ MRC-5 cells with HSV-1 at an MOI of 10 in the presence of the indicated concentrations of inhibitor. Virus was harvested 24 hpi and titers were determined by plaque assay on Vero cells. Data represent the means of biological duplicate experiments with standard deviations. (E) One million MRC-5 cells were infected at an MOI of 0.1 or 10 PFU/cell in the presence or absence of SP-2509 (16 μM) and virus was collected at indicated times. Viral yield was measured via plaque assay on Vero cells. All values represent the means of biological duplicate experiments with standard deviations. Data points without error bars represent reproducible data and error bars that were smaller than the symbol used to represent the data point.

The observation that LSD1 modifies histone H3 associated with infecting viral DNA early during infection (33, 35), but stably associates with nascently replicated viral DNA (22, 26), suggests that it may play an additional role later during infection. To address the effects of LSD1 inhibition on viral processes, we first determined the cytotoxic effects of the LSD1 inhibitors SP-2509 and OG-L002 in MRC-5 and Vero cells using an MTT colorimetric assay (Fig. 1A and B). Subconfluent cells were incubated in the presence of SP-2509 or OG-L002 for 24 h before performing an MTT assay. Significant cytotoxicity was observed above 30 μM SP-2509 in both cell lines (Fig. 1A). OG-L002 was cytotoxic to MRC-5 cells at 120 μM (Fig. 1B). Using the concentrations tested in this assay, we did not observe cytotoxicity after treatment of Vero cells with OG-L002.

We next assayed the effects of SP-2509 and OG-L002 on viral yield (Fig. 1C and D). MRC-5 or Vero cells were infected with HSV-1 strain KOS at a multiplicity of infection (MOI) of 10 in the presence or absence of increasing concentrations of SP-2509 or OG-L002. After 24 h, virus was harvested and viral yield was determined by plaque assay in Vero cells. Treatment with SP-2509 resulted in about a 130,000-fold reduction in viral yield compared to dimethyl sulfoxide (DMSO)-treated controls, while treatment with OG-L002 resulted in a 7.2 to 19-fold reduction at the concentrations tested. Higher concentrations of inhibitors were not tested because they are cytotoxic (Fig. 1A and B). Previously it was shown that treatment of HFF cells with 50 μM OG-L002 results in a 100-fold reduction in viral yield compared to an untreated control after a 24-h low multiplicity infection (0.1 PFU/cell) (35). Here, effects of SP-2509 inhibition were also amplified at low multiplicity (Fig. 1E). Taken together, these results indicate that SP-2509 is a novel and potent inhibitor of HSV-1 infection.

### SP-2509 inhibits late protein expression.

**FIG 2:**
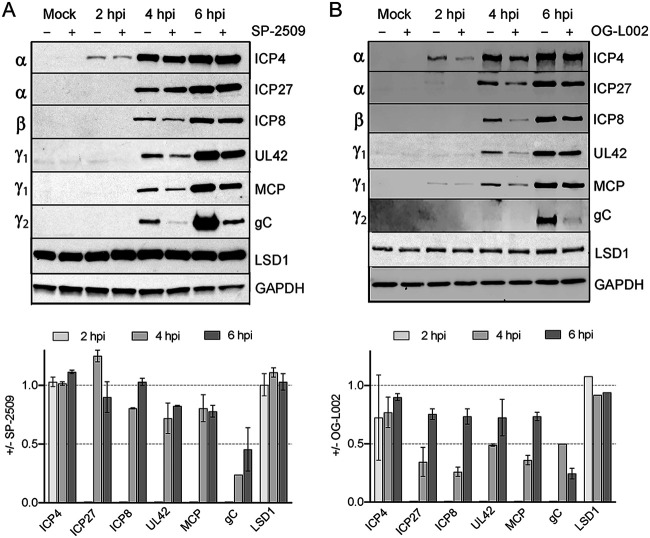
Effects of LSD1 inhibition on viral protein expression. (A) Western blot of whole-cell lysates collected from SP-2509 treated (16 μM) or untreated MRC-5 cells mock infected or infected with strain KOS and harvested at 2, 4, and 6 hpi. Antibodies are indicated on the right and the viral gene class is indicated on the left. (B) Experiments were carried out as in (A) except that cells were treated with or without OG-L002 (60 μM), infected with strain KOS, and harvested at the indicated times. All infections were carried out at an MOI of 10 PFU/cell and for all inhibitor treatments cells were pretreated with inhibitor for 1 h prior to infection and throughout infection. Average fold change in protein expression (+inhibitor/−inhibitor) was determined from biological duplicate experiments and error bars represent standard deviations.

We next examined the effects of SP-2509 treatment on viral protein expression. MRC-5 cells were infected with HSV-1 at an MOI of 10 PFU/cell in the presence or absence of 16 μM SP-2509. The previously determined *in vitro* 50% inhibitory concentration (IC_50_) for SP-2509 is 12.6 μM (38) and previous *in vivo* studies were carried out using up to 10 μM SP-2509 (37). Total proteins were isolated at 2, 4, or 6 h postinfection (hpi) and viral and cellular protein levels were determined by Western blotting (Fig. 2A). Levels of individual proteins were normalized to glyceraldehyde 3-phosphate dehydrogenase (GAPDH) expression and the fold change in expression in the presence versus absence of SP-2509 was determined. Although SP-2509 treatment caused a significant reduction in viral yield, it had little to no effect on IE (α: ICP4, ICP27) or early (β: ICP8) protein levels. On the other hand, there was a moderate effect on leaky late (γ1: UL42, major capsid protein [MCP]) and a significant reduction in late (γ2: gC) protein levels. Consistent with previous observations (35), treatment with 60 μM OG-L002 resulted in reduced expression of viral proteins expressed from all classes of viral genes (Fig. 2B). Neither inhibitor caused altered levels of LSD1. Taken together, these data suggest that although OG-L002 and SP-2509 both inhibit HSV-1 infection, they may do so through different mechanisms.

### SP-2509 inhibits replication-dependent transcription from HSV-1 genomes.

**FIG 3:**
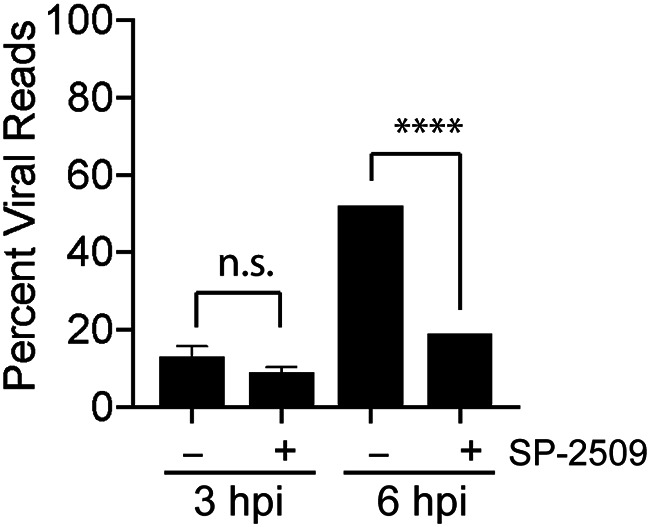
SP-2509 inhibits viral gene expression. MRC-5 cells were infected at an MOI of 10 PFU/cell with strain KOS in the presence or absence of SP-2509 (16 μM) and RNA-seq was carried out on total mRNA at 3 and 6 hpi. Sequencing reads were mapped to the human and HSV-1 genome and the percentage of RNA-seq reads that mapped to the viral genome was determined. Data represent the means from biological duplicate experiments with standard deviations. For the 6 hpi time points, results from replicate experiments were identical. Therefore, error bars are not shown on the bar graph. The significance of differences observed in the presence of SP-2509 were determined using a Student’s *t* test with a statistical significance cutoff at 0.05 (n.s, not significant; ****, *P* < 0.0001).

To determine how the temporal expression of viral genes is globally affected by SP-2509 treatment, we measured viral mRNA levels in the presence and absence of SP-2509 at an early (3 h) and late (6 h) time after infection using RNA sequencing (RNA-seq). MRC-5 cells were pretreated with 16 μM SP-2509 for 1 h before infection. During high MOI infection (10 PFU/cell), IE and early genes are expressed and viral DNA begins to replicate by 3 hpi (26, 39). By 6 hpi, late gene expression predominates, while several IE and early viral genes are also amplified in a replication-dependent manner (39). At 3 hpi, under untreated infection conditions, 13% of the sequence reads mapped to the HSV-1 genome, while 83% mapped to the cellular genome, and the remaining 4% did not map (Fig. 3A). At 3 hpi, SP-2509 did not cause a significant change in viral gene expression. However, by 6 hpi, SP-2509 treatment resulted in a significant decrease in viral gene expression. At this time, viral mRNA predominates and makes up over 50% of transcripts per cell. SP-2509 treatment caused a drop in percent viral reads by 31%, demonstrating a significant block in viral gene expression late after infection.

**FIG 4:**
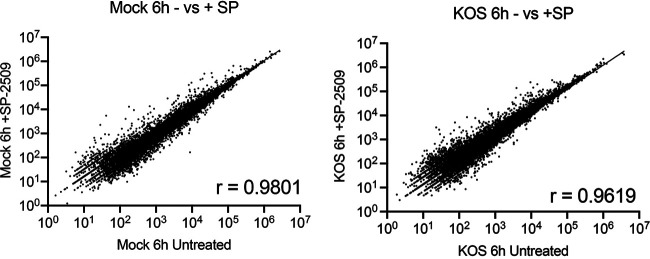
Cellular mRNA expression is not significantly altered by SP-2509 treatment. RNA sequencing reads were mapped to the human genome and mapped reads to individual cellular transcripts were compared between the indicated conditions. The value *r* represents the Pearson correlation between data sets.

To determine if SP-2509 treatment altered cellular gene expression in these experiments, we compared the relative expression levels of individual cellular transcripts in mock-infected cells in the presence and absence of SP-2509 (Fig. 4, left). These data sets had a high Pearson correlation coefficient (*r*) of 0.98. The same was true when comparing the cellular gene expression profiles of HSV-1 infected cells in the presence and absence of inhibitor (Fig. 4, right; *r* = 0.96). Therefore, at the concentrations used in this study and during the first 6 to 7 h after treatment, SP-2509 does not significantly alter the cellular gene expression profile.

**FIG 5:**
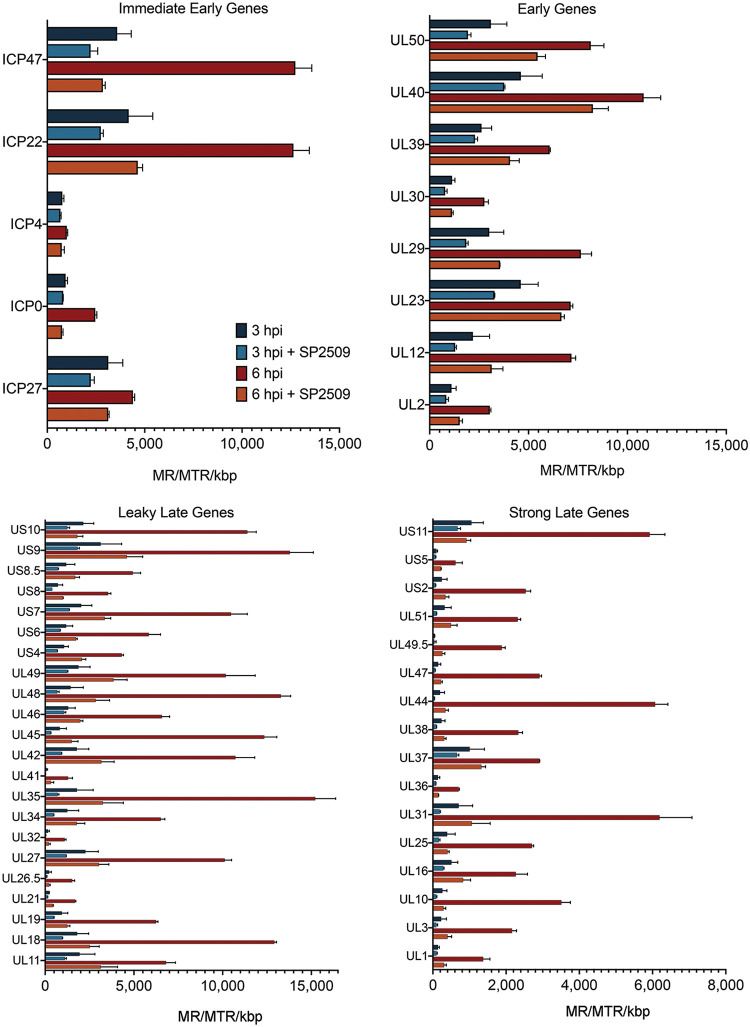
Abundance of individual viral transcripts at 3 and 6 hpi in the presence or absence of SP-2509 during high multiplicity infection. The abundance of individual viral mRNAs is presented as mapped reads per mapped total reads per kilobase pair (MR/MTR/kbp). Error bars represent the standard deviation from the mean of two biological replicates. Transcripts are grouped based on gene class (39). To determine if changes observed when comparing the expression of each gene from samples infected in the presence versus absence of SP-2509 were significant, we used a Student’s *t* test with a statistical significance cutoff of 0.05. At 3 hpi, SP-2509 did not have a statistically significant effect on the expression of individual viral genes. At 6 hpi, differences in the levels of expression of all transcripts were statistically significant except for ICP4, ICP27, and UL23.

We next determined how expression of individual viral genes is affected by SP-2509 treatment. Genes were grouped into IE, early, leaky late, and late gene classes and mRNA levels were compared in the presence and absence of SP-2509 at 3 and 6 hpi (Fig. 5). At 3 hpi, there were no significant differences in the levels of expression of any gene class (*P* > 0.05). Most late genes were barely detected above background levels at this time. By 6 hpi, expression of all viral genes were amplified with the exception of ICP4 and ICP27 (Fig. 5, compare 3 and 6 hpi). This is likely because more viral genome templates are present after the DNA is replicated. These same genes exhibited decreased expression at 6 hpi in the presence of SP-2509 (Fig. 5). Late gene expression was significantly reduced, in most cases to almost undetectable levels, in the presence of SP-2509. Taken together, while initial activation of transcription was not significantly affected by SP-2509 treatment, replication-dependent late gene expression was inhibited and replication-coupled amplification of IE, early, and late gene transcription was significantly reduced.

### SP-2509 inhibits viral DNA replication.

**FIG 6:**
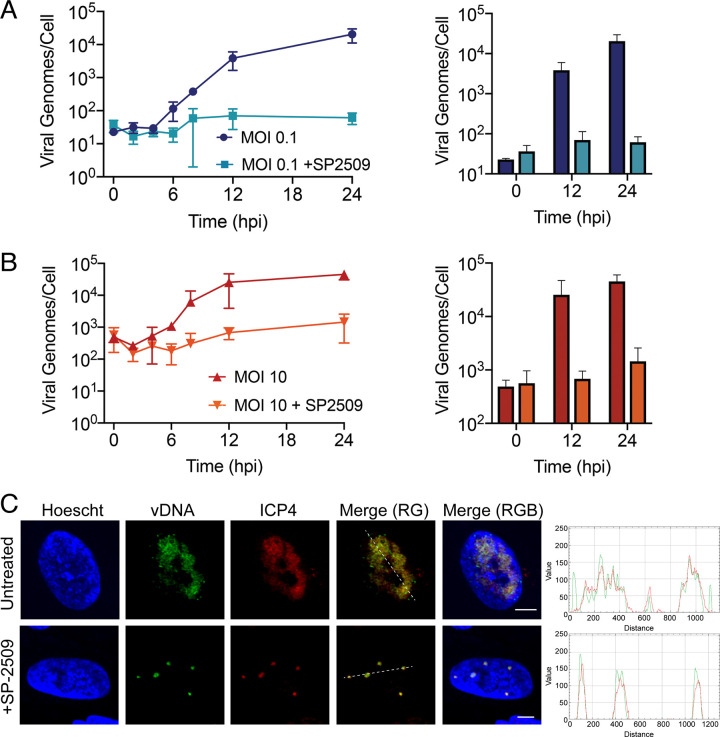
SP-2509 blocks HSV-1 DNA replication. (A and B) MRC-5 cells were infected at an MOI of 0.1 (A) or 10 (B) PFU/cell with strain KOS in the presence or absence of SP-2509 (16 μM). DNA was collected at the indicated times to create a replication curve. Viral genome number was determined by real-time PCR. Error bars represent the means with standard deviations. Data points without error bars represent reproducible data and error bars that were smaller than the symbol used to represent the data point. (C) MRC-5 cells were infected at an MOI of 10 PFU/cell with strain KOS in the presence or absence of SP-2509 (16 μM). EdC was added at 4 hpi, and at 6 hpi cells were fixed. Viral genomes were covalently attached to a fluorophore (vDNA) and probed with antibodies specific to ICP4. Scale bars, 5 μm. Green and red trace of merge (RG) panel is shown at right.

RNA-seq data point to a defect in replication-coupled viral gene expression. Therefore, we determined the effect of SP-2509 treatment on viral DNA synthesis. To do this, we collected viral DNA from infected MRC-5 cells at multiple times after infection in the presence or absence of 16 μM SP-2509 and measured viral DNA levels using quantitative real-time PCR (Fig. 6A and B). At both low (0.1 PFU/cell) and high (10 PFU/cell) MOIs, SP-2509 had a potent and reproducible inhibitory effect on viral DNA replication.

Real-time PCR data suggest that low levels of DNA replication can still occur in the presence of SP-2509. To verify that viral DNA replication initiates in the presence of SP-2509, we imaged nascent viral DNA in the presence of the drug. MRC-5 cells were infected with strain KOS at an MOI of 10 PFU/cell in the presence or absence of SP-2509 and incubated in medium containing 5-ethynyl-2′-deoxycytidine (EdC) from 4 to 6 hpi to label nascent viral DNA. We then tagged and imaged the EdC-labeled DNA and demonstrated that EdC incorporation and some viral DNA replication does occur in the presence of SP-2509 and that ICP4 colocalizes with the replicated population of viral DNA (Fig. 6C).

**FIG 7:**
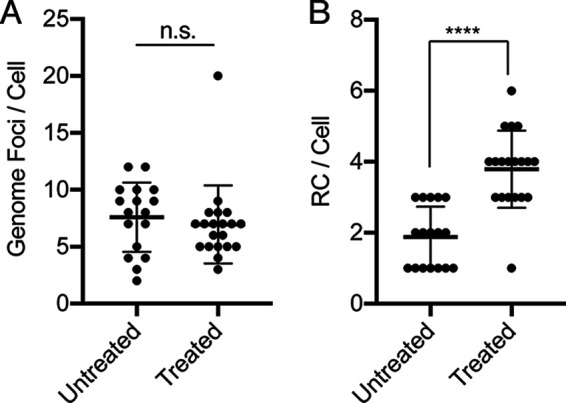
SP-2509 treatment has no effect on viral genome entry into the nucleus, but results in an increased number of replication compartments per cell. (A) MRC-5 cells were infected with KOS-EdC at an MOI of 10 PFU/cell in the presence or absence of SP-2509 (16 μM) and fixed at 2.5 hpi. Input viral DNA was covalently attached to Alexa Fluor 488 and imaged. The number of input viral genomes per cell was counted. (B) MRC-5 cells were infected with strain KOS at an MOI of 10 PFU/cell in the presence or absence of SP-2509 (16 μM). EdC was added at 4 hpi, and at 6 hpi the cells were fixed. EdC-labeled replicated viral DNA was covalently attached to Alexa Fluor 488 and imaged. The number of viral replication compartments per cell was counted. Horizontal lines in the scatter dot plot represent the means with standard deviations. Significance was determined using a Student’s *t* test with a statistical significance cutoff of 0.05 (n.s, not significant; ****, *P* < 0.0001).

On average, 3 to 5 small replication foci formed in the presence of inhibitor (+SP-2509) compared to the 1 to 3 large replication compartments that formed in infected, untreated cells (Fig. 7A). The increased number of replication compartments in the presence of SP-2509 suggests that replication is blocked before intergenomic recombination can occur. After an MOI of 10 PFU/cell infection with EdC-labeled strain KOS, incoming viral DNA forms 5 to 10 viral DNA foci in the presence or absence of SP-2509 (Fig. 7B). It has previously been demonstrated that only a fraction of incoming viral genomes initiate replication during infection with HSV-1 (40). This is consistent with the presence of more incoming viral genomes compared to replication foci in the presence of SP-2509. Taken together, we conclude that SP-2509 inhibits some step in viral infection after the initial onset of viral DNA synthesis, likely before replication compartments coalesce.

### SP-2509 selectively and reversibly inhibits viral DNA replication.

**FIG 8:**
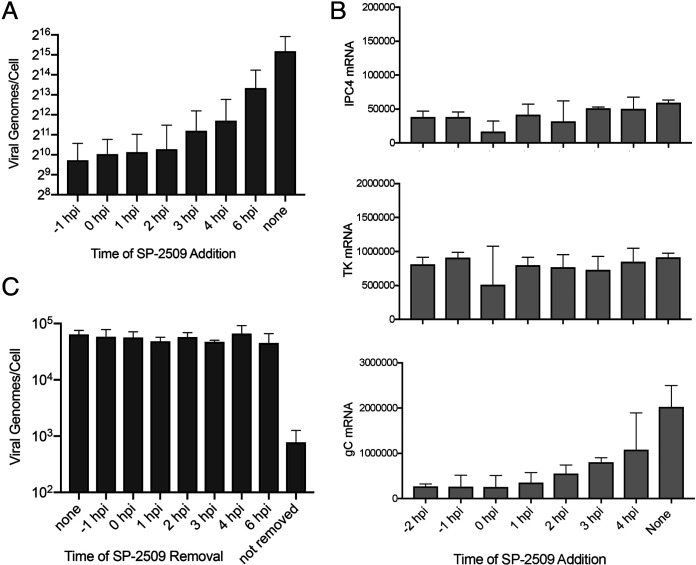
SP-2509 inhibits viral DNA replication and late gene expression if added postinfection and is reversible. (A) MRC-5 cells were infected at an MOI of 10 PFU/cell and SP-2509 (16 μM) was added at the indicated times before or during viral infection. Viral genome number was quantified by real-time PCR to amplify the tk gene from DNA isolated at 12 hpi. (B) Infection was carried out as in (A) except that RNA was isolated at 6 hpi, reverse transcribed, and amplified by real-time PCR to determine the relative expression of the ICP4 (α), tk (β), or gC (γ) genes. (C) SP-2509 was removed from cells at the indicated times relative to the start of infection. DNA was collected at 12 hpi and quantified as in (A). All experiments were carried out in biological duplicate and error bars represent the standard deviation from the mean.

To verify that early events in the infection cycle do not contribute to the block in viral DNA replication, we determined the effect of time of SP-2509 addition on viral DNA replication (Fig. 8A). Cells were infected with strain KOS at an MOI of 10 PFU/cell and SP-2509 was added either prior to (−1 h) or at 0, 1, 2, 3, 4, or 6 hpi. The number of viral genomes present per cell at 12 hpi was determined by quantitative real-time PCR. Viral DNA replication was negatively impacted by SP-2509 regardless of the time of addition. This indicates that under conditions where IE and early genes are expressed in an appropriate temporal manner, SP-2509 can still potently inhibit viral DNA replication.

We next investigated the effect of time of SP-2509 addition on viral transcription by assaying mRNA levels of select viral transcripts at 6 hpi by reverse transcription and real-time PCR (Fig. 8B). Transcription of the late viral gene, gC (UL44), was affected by SP-2509 addition in a similar manner as viral genome replication. On the other hand, expression of the IE gene ICP4 and early gene tk (UL23) were not affected by the addition of SP-2509. These results clearly demonstrate that SP-2509 selectively blocks viral DNA replication and replication-coupled late gene expression.

Furthermore, because SP-2509 inhibition is reversible, we determined if the block in viral DNA replication can be reversed after SP-2509 removal. If so, this may be a unique approach to synchronize viral replication forks to investigate the coordination of replication-coupled events in the future. To determine if the block in viral DNA replication can be reversed after SP-2509 removal, we infected cells at an MOI of 10 PFU/cell in the presence of SP-2509 and removed the inhibitor at the indicated times during infection (Fig. 8C). The number of viral genomes present per cell at 12 hpi was then measured by quantitative real-time PCR. In all cases, removal of SP-2509 fully restored the capacity for viral DNA to replicate to levels comparable to untreated cells, demonstrating that SP-2509 is a reversible inhibitor of viral DNA replication.

### SP-2509 does not inhibit nuclear entry or early interactions that occur on viral DNA.

**FIG 9:**
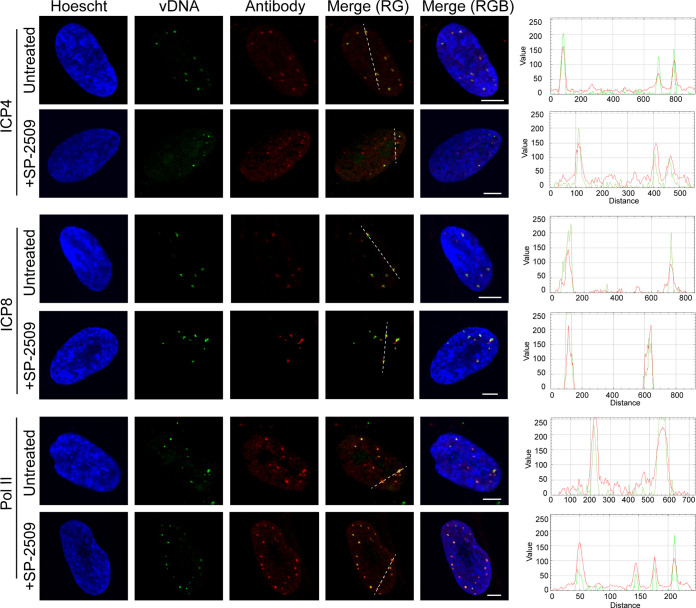
SP-2509 treatment does not affect nuclear entry or genome interactions during early infection. MRC-5 cells were infected at an MOI of 10 PFU/cell with EdC-labeled KOS in the presence or absence of SP-2509. At 2.5 hpi, cells were fixed and viral genomes were covalently attached to a fluorophore (vDNA) and probed with antibodies specific for ICP4, ICP8, or Pol II. Scale bars, 5 μm. Green and red trace of merge (RG) panel is shown at right.

To further verify that SP-2509 does not inhibit early processes that occur on viral DNA, viral genome entry into the nucleus and initial recruitment of viral and host cell factors to incoming viral DNA were examined. MRC-5 cells were infected with EdC-labeled strain KOS at an MOI of 10 PFU/cell and fixed at 2.5 hpi. EdC-labeled viral DNA was covalently attached to Alexa Fluor 488 and viral and host proteins were detected by indirect immunofluorescence (Fig. 9). SP-2509 treatment had no observable effect on viral genome entry into the nucleus (Fig. 7B). Furthermore, colocalization of the viral transcription factor ICP4, the viral replication protein ICP8, and Pol II with incoming viral DNA were not affected by SP-2509 by 2.5 hpi.

### Replication proteins colocalize with viral DNA in the presence of SP-2509.

**FIG 10:**
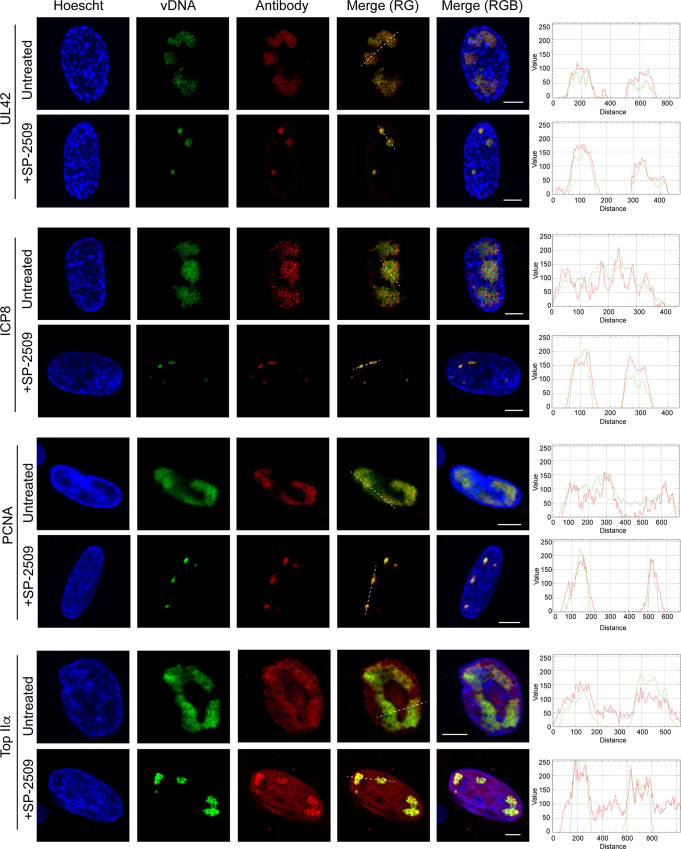
SP-2509 treatment results in defective viral replication compartment formation, but key replication-dependent proteins maintain their ability to colocalize with viral DNA. MRC-5 cells were infected at an MOI of 10 PFU/cell with KOS in the presence or absence of SP-2509 (16 μM). EdC was added at 4 hpi, and at 6 hpi cells were fixed. EdC-labeled viral DNA was covalently attached to a fluorophore (vDNA) and probed with antibodies specific to viral proteins UL42 and ICP8, and cellular proteins PCNA and topoisomerase IIα. Scale bars, 5 μm. Green and red trace of merge (RG) panel is shown at right.

We next examined whether viral and cellular replication proteins colocalize with EdC-labeled viral replication compartments in the presence of SP-2509 (Fig. 10). Replicating viral DNA was labeled with EdC and imaged as in Fig. 6C. Using immunofluorescence, we demonstrate that the viral DNA processivity factor (UL42) and single-stranded DNA binding protein (ICP8) colocalize with replication compartments that form in SP-2509-treated cells. The cellular replication proteins PCNA and topoisomerase IIα (TOP IIα) also associate with these sites of defective DNA synthesis.

**FIG 11:**
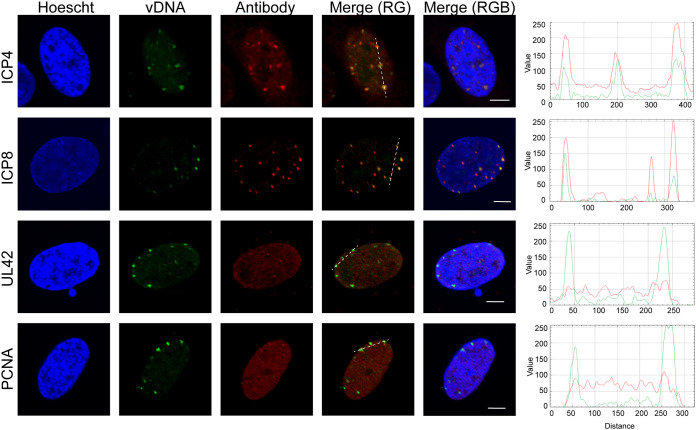
Protein recruitment to viral DNA after ACV treatment. Cells were infected with EdC-labeled KOS at an MOI of 10 PFU/cell in the presence of 100 μM ACV. After 6 h, infected cells were fixed and EdC-labeled DNA was tagged with an Alexa fluor to visualize viral genomes (vDNA). Viral and cellular proteins were visualized by immunofluorescence. Green and red trace of merge (RG) panel is shown at right. Scale bars, 5 μm.

We next compared the effects of SP-2509 treatment on the recruitment of proteins to viral DNA to that of acyclovir (ACV) treatment. Cells were infected with EdC-labeled strain KOS in the presence of ACV and at 6 hpi cells were fixed and imaged. By comparison, UL42, PCNA, and TOP IIα do not colocalize with viral genomes after ACV treatment, although ICP4 and ICP8 do (Fig. 11). Taken together, these data suggest that SP-2509 blocks viral DNA replication at a step later than ACV treatment, after viral and cellular replication proteins are recruited to viral replication forks. The association of ICP8 (single-stranded DNA binding protein) and ICP4 (double-stranded DNA binding protein) with the aberrant replication compartments that form during SP-2509 treatment is consistent with the viral DNA containing both single- and double-stranded regions.

### SP-2509 selectively inhibits protein binding after the onset of viral DNA replication.

**FIG 12:**
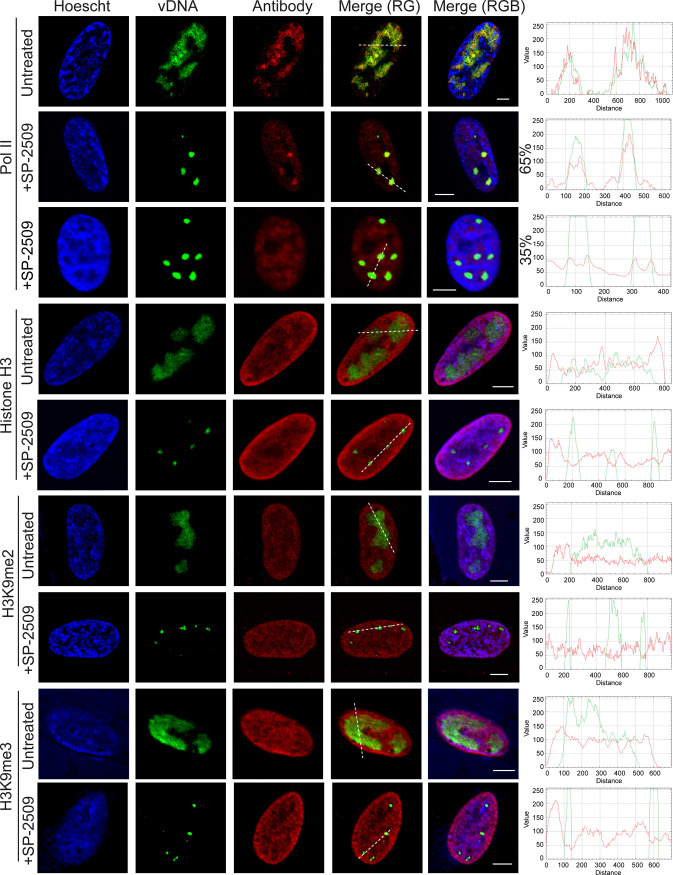
Recruitment of Pol II to viral DNA is defective in the presence of SP-2509. MRC-5 cells were infected at an MOI of 10 PFU/cell with strain KOS in the presence or absence of SP-2509 (16 μM). EdC was added at 4 hpi and cells were fixed at 6 hpi. Viral genomes were covalently attached to a fluorophore (vDNA) and probed with antibodies specific for the indicated proteins. For Pol II, two different colocalization phenotypes were observed. The top row (with SP-2509) represents the colocalization of Pol II with replicated viral DNA, which occurred in 33 out of 51 counted cells (65%), while the bottom row represents the lack of colocalization of replicated viral DNA with Pol II, which occurred in 18 out of 51 counted cells (35%). Scale bars, 5 μm. Green and red trace of merge (RG) panel is shown at right.

Because there was a defect in viral transcription after the onset of viral DNA replication in the presence of SP-2509, we next examined whether Pol II was recruited to replicated viral DNA in the presence of SP-2509 (Fig. 12). We found that in about 65% of cells, Pol II colocalized with EdC-labeled viral replication compartments in treated cells in a similar manner as in untreated cells. However, in another 35% of infected cells, Pol II did not colocalize with EdC-labeled viral DNA. How the state of the cell contributes to these distinct phenotypes is of interest for future investigation. We further demonstrate that the localization of histone H3, H3K9me2, and H3K9me3 relative to replicated viral DNA is similar between treated and untreated cells. Taken together, although observable changes in histone localization do not occur at late times during infection in the presence of SP-2509, Pol II recruitment to viral DNA is defective.

## DISCUSSION

Here, we demonstrate that SP-2509 is a potent and reversible inhibitor of HSV-1 infection. SP-2509 inhibits viral DNA replication and replication-coupled gene expression, but does not affect viral genome entry into the nucleus and protein association with viral DNA to facilitate IE and early gene transcription. Furthermore, in the presence of SP-2509, viral DNA begins to replicate but ongoing DNA replication is inhibited. Stalled DNA synthesis in the presence of SP-2509 does not impede replication protein association but does result in altered replication compartment formation and recruitment of Pol II to viral DNA. Taken together, SP-2509 is a new inhibitor of HSV-1 infection that blocks a critical step during HSV-1 DNA replication.

Observations presented here support a potential new role for LSD1 in promoting viral DNA replication and replication-dependent viral gene expression. A role for LSD1 at sites of viral DNA synthesis is supported by the observation that LSD1 associates with viral replication forks and nascent viral DNA (7, 26). It was previously demonstrated that LSD1 knockdown by small interfering RNA (siRNA) and LSD1 inhibitors TCP and OG-L002 block H3K9 demethylation on promoters of IE genes during early stages of infection, resulting in a reduction of IE gene expression (33, 35, 41). We did not observe the same effect on IE gene expression when infection was carried out in the presence of SP-2509. TCP and its derivative OG-L002 inhibit LSD1 by covalently attaching to the cofactor FAD bound to the active site of the enzyme, thereby irreversibly inhibiting demethylase activity. SP-2509 has been shown to inhibit LSD1 demethylase activity *in vitro* and disrupt LSD1 protein-protein interactions *in vivo*, including interactions with ZNF217 (37) and CoREST (36). It is therefore possible that SP-2509 inhibits LSD1 transcription factor interactions that are important at late stages of infection rather than histone demethylation, which is necessary for IE gene expression. It is possible that differences in the mechanisms of action of these different drugs account for the differential effects on HSV-1 infection. An alternative hypothesis is that SP-2509 inhibits viral infection through a completely different mechanism that does not involve LSD1. The precise mechanism of SP-2509 inhibition is of interest for future studies.

During high multiplicity infection (10 PFU/cell), we observed 5 to 10 input viral DNA foci in the nuclei of infected cells even in the presence of SP-2509. Under normal infection conditions, several of these genomes undergo DNA replication and coalesce to form 1 to 3 large replication compartments. Although SP-2509 inhibits viral DNA replication, 3 to 5 small replication compartments form during infection in treated cells. However, these small replication foci do not coalesce to form large replication compartments. These data suggest that in the presence of SP-2509, intergenomic recombination is at least partially inhibited. HSV-1 DNA replication occurs through two phases. Initially replication occurs through an origin-primed process involving the actions of UL9 and ICP8 at one of three origins of replication (42). However, later during infection, replication is thought to switch to a recombination-based mechanism (8). Perhaps LSD1 plays a role in mediating this switch.

We previously demonstrated that initial rounds of viral DNA replication are sufficient to license late gene expression (26). Furthermore, ongoing expression of ICP4 is required for continuous expression of late genes, even in the presence of robust DNA replication (39). In the presence of SP-2509, initial replication can occur, however, late gene expression is inhibited, decoupling this licensing event in a similar manner as ICP4 depletion. These data point to a potential function of LSD1 in the replication-coupled regulation of late gene expression, consistent with aberrant recruitment of Pol II to viral genomes in the presence of SP-2509.

Tranylcypromine (TCP) is a potent anti-HSV inhibitor in mouse, rabbit, and guinea pig model systems (34). TCP is an irreversible and nonselective monoamine oxidase (MAO) inhibitor that targets MAO-A, MAO-B, and LSD1 (35). In contrast, SP-2509 is over 7,000-fold more selective for LSD1 over MAO-A and MAO-B and is reversible (36). SP-2509 has been investigated for use as an anti-cancer drug against cancers with high LSD1 expression and poor prognosis, including acute myeloid leukemia (36), Ewing sarcoma (43), and advanced prostate cancer (37). Therefore, SP-2509 or its derivatives may be good candidates for antiviral therapy in the future, especially to treat ACV-resistant strains. Together with other previous studies, these data highlight the potential to utilize LSD1 inhibition as an antiviral approach to treat HSV-1 infection.

## MATERIALS AND METHODS

### Cells and viruses.

MRC-5 (human fetal lung fibroblast) or Vero (African green monkey kidney) cells were obtained from and propagated as recommended by ATCC. HSV-1 strain KOS was used for all infections.

### MTT assay.

An MTT assay (Abcam) was carried out to measure the effects of LSD1 inhibitors on cell viability and proliferation. Cells were plated in 96-well plates for 24 h prior to addition of inhibitors (1.0 × 10^4^ cells/well). The indicated concentrations of SP-2509 or OG-L002 were added and cells were incubated at 37°C for an additional 24 h before conducting the MTT assay according to the manufacturer’s protocol. Percent cytotoxicity was determined: $\%\text{ Cytotoxicity = }\frac{\text{(100 }x\text{ (}\mathrm{Control}-\mathrm{Sample}\text{))}}{\mathrm{Control}}$.

### Viral yield.

MRC-5 or Vero cells were seeded at a density of 1 × 10^6^ cells/well in a 6-well dish and infected with strain KOS at an MOI of 0.1 or 10 PFU/cell. Infected treated cells were incubated in medium containing the indicated concentration of SP-2509 or OG-L002 or a DMSO control. At the indicated times, infected cells were collected by scraping into growth medium and freeze-thawed 3 times, followed by sonication to release cell-associated virus. Viral yield was determined by plaque assay in Vero cells.

### Western blotting.

MRC-5 cells were seeded at a density of 1 × 10^6^ cells/well in a 6-well dish and infected with strain KOS at an MOI of 10 PFU/cell. Treated cells were incubated with 16 μM SP-2509 or 60 μM OG-L002 for 1 h prior to and during infection. At the indicated times postinfection, proteins were isolated from cells using Laemmli SDS sample buffer and Western blotting was carried out using the following primary antibodies: α-ICP4 (58S), α-ICP27 (P1113), α-ICP8 (Abcam ab20194), α-UL42 (Abcam ab19311), α-VP5/ICP5 (Abcam ab6508), α-gC (GICR 1104), α-GAPDH (Thermo AM4300), and α-LSD1 (Abcam ab37165). The intensity of gel bands was quantified using the GelAnalyzer plugin in ImageJ. Band intensities were normalized to GAPDH detected from the same sample.

### RNA-seq.

MRC-5 cells were seeded into 60-mm dishes at a density of 2 × 10^6^ cells per dish. Treated cells were pretreated with 16 μM SP-2509 for 1 h prior to infection with strain KOS at an MOI of 10 PFU/cell or mock infection and incubated at 37°C. At 3 or 6 hpi, RNA was isolated using the RNaqueous-4 PCR kit (Invitrogen) using the included protocol. The isolated total RNA was quantified using an RNA 6000 Nano kit (Agilent) and a 2100 Bioanalyzer (Agilent). Barcoded cDNA libraries were prepared from 2 μg of total RNA using the NEBNext Poly(A) mRNA Isolation and NEBNext Ultra Directional RNA library prep kit. Individual samples were quantified using a DNA 7500 kit (Agilent) and 2100 Bioanalyzer (Agilent). Barcoded samples were pooled and sequenced at the Tufts University genomics facility on a HiSeq 2500 (Illumina). The demultiplexed sequence reads were first mapped to the human reference genome (hg38) using HISAT2. Unmapped reads were then mapped to a modified version of the annotated KOS genome (KT899744) excluding duplicated regions of the genome (nucleotides 1 to 9063 containing the RL1 and RL2 gene regions and nucleotides 145359 to 151974 containing the RS1 gene). featureCounts was used to quantify transcripts. Mapped reads of individual transcripts were normalized to account for differences in size and sequencing depth and are presented as mapped reads per million total reads per kilobase pair (MR/MTR/kbp). For all conditions examined, data are highly reproducible between biological duplicates with Pearson correlation coefficients of >0.90.

### Viral DNA replication curve.

MRC-5 cells were seeded at 1 × 10^6^ cells/well in a 6-well dish. Treated cells were incubated with 16 μM SP-2509 1 h before infection and postinfection. Cells were infected with strain KOS at an MOI of 0.1 or 10 PFU/well. DNA was isolated using DNA extraction buffer (0.5% SDS, 400 μg/ml proteinase K, 100 mM NaCl). Genome numbers were determined by real-time PCR relative to a standard curve generated from purified viral DNA. Primers specific for the tk gene (tkdsf 5′-ACCCGCTTAACAGCGTCAACA-3′, tkdsr 5′-CCAAAGAGGTGCGGGAGTTT-3′) were used for viral DNA amplification. Cell numbers were determined by amplification of cellular DNA using primers specific for GAPDH (GAPDHf 5′-CAGAACATCATCCCTGCCTCTACT-3′, GAPDHr 5′-GCCAGTGAGCTTCCCGTTCA-3′). Two biological replicates were carried out for each condition.

### Immunofluorescence and viral DNA imaging.

A total of 2 × 10^5^ MRC-5 cells were grown on glass coverslips and infected with wild-type KOS or EdC-labeled KOS at an MOI of 10 PFU/cell for 2.5 or 6 h in the presence or absence of 16 μM SP-2509. EdC labeling of viral replication compartments, click chemistry, and immunofluorescence were conducted as previously described (22). Primary antibodies used include: α-ICP8 (Abcam ab20194), α-ICP4 (58S), α-RNA polymerase II (Abcam ab5408), α-topoisomerase II (Calbiochem Ab-1 NA14), α-PCNA (Santa Cruz sc-056), α-Histone H3 (Abcam ab1791), α-UL42 (Abcam ab19311), α-H3K9me2 (Abcam ab1220), and H3K9me3 (Abcam ab176916). Relative fluorescence intensities were plotted using the RGB Profiler plugin for ImageJ.

### Quantification of viral gene expression by real-time PCR.

MRC-5 cells were seeded at 1 × 10^6^ cells/well in a 6-well dish. Cells were infected with strain KOS at an MOI of 10 PFU/cell. Treated cells were incubated with 16 μM SP-2509 before and during infection as indicated. At 6 hpi, RNA was isolated using the RNAqueous kit 4PCR (Invitrogen) followed by reverse transcription using the MMLV HP reverse transcriptase (Epicentre) and an oligo(dT) primer (IDT) according to the manufacturer’s protocol. Viral genes were amplified by real-time PCR using the following primers: ICP4 (5′-CCACGGGCCGCTTCAC-3′, 5′-GCGATAGCGCGCGTAGAA-3′), tk (5′-ACCCGCTTAACAGCGTCAACA-3′, 5′-CCAAAGAGGTGCGGGAGTTT-3′), and gc (5′-GTGACGTTTGCCTGGTTCCTGG-3′, 5′-GCACGACTCCTGGGCCGTAACG-3′).

### Data availability.

The RNA-seq data sets are available in the SRA database under accession number PRJNA562764.
